# The serum creatinine to cystatin C to waist circumference ratios predicts risk for type 2 diabetes: A Chinese cohort study

**DOI:** 10.1111/1753-0407.13435

**Published:** 2023-07-05

**Authors:** Yinfei Chen, Weiheng Wen, Zhiliang Mai, Ming Wang, Hong Chen, Jia Sun

**Affiliations:** ^1^ Department of Endocrinology, Zhujiang Hospital Southern Medical University Guangzhou China; ^2^ Department of Digestive Medicine, Zhujiang Hospital Southern Medical University Guangzhou China; ^3^ Department of Traditional Chinese Medicine, Zhujiang Hospital Southern Medical University Guangzhou China; ^4^ Present address: Shangyu People's Hospital of Shaoxing Shaoxing China

**Keywords:** creatinine, cystatin C, sarcopenic obesity, type 2 diabetes, waist circumference, 肌酐, 胱抑素C, 腰围, 2型糖尿病, 肌肉减少性肥胖

## Abstract

**Background:**

There is a lack of research regarding the relationship between creatinine to cystatin C to waist circumference ratio (CCR/WC ratios) and the development of type 2 diabetes mellitus (T2DM). We aimed to evaluate the association between CCR/WC ratios and incident T2DM in Chinese adults.

**Methods:**

This prospective study was from the China Health and Retirement Longitudinal Study (2011, 2013, 2015, and 2018). The participants were divided into three groups by tertiaries of the CCR/WC ratios. Cox proportional‐hazards models were used to identify the relationship between CCR/WC and T2DM.

**Results:**

Overall, 5938 participants were included for analysis, 766 of whom developed T2DM between 2011 and 2018. Risk of incident T2DM was decreased with tertiaries 2, 3 versus tertiary 1 of the CCR/WC index (adjusted hazard ratio [HR] 0.772 [95% confidence interval 0.647–0.921] and 0.724 [0.596–0.880], *p* for trend = .001 across tertiaries of the CCR/WC index). The results were consistent excluding participants with T2DM in the first 2 years.

**Conclusions:**

This study demonstrated that CCR/WC was negatively correlated with the risk of T2DM in Chinese adults. Early detection is necessary to control the development of T2DM in Chinese with low CCR/WC levels.

## Graphical abstract

**Highlights**

- Higher creatinine to cystatin C to waist circumference ratio (CCR/WC ratio) was associated with decreased risk of type 2 diabetes mellitus (T2DM) among Chinese people.
- A nonlinear association was found between CCR/WC and T2DM risk.

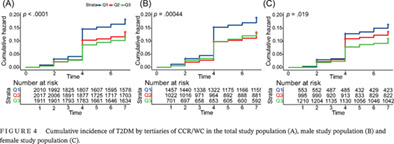

## 摘要

背景: 关于肌酐/胱抑素C与腰围比值（CCR/WC）与2型糖尿病（T2DM）发病之间的关系，目前尚缺乏研究。探讨中国成年人CCR/WC比值与2型糖尿病发病风险的关联。

方法: 这项前瞻性研究来自中国健康与退休纵向研究（2011年、2013年、2015年和2018年）。参与者根据CCR/WC的比例被分为三组。使用Cox比例风险模型确定CCR/WC与T2DM之间的关系。

结果: 有5938名参与者被纳入分析，其中766人在2011年至2018年间发展为T2DM。与CCR/WC指数最低三分位数相比，第二和第三三分位数与T2DM的发病风险降低相关（调整后HR分别为0.772 [95% CI 0.647‐0.921]和0.724 [0.596‐0.880]，P值趋势=0.001）。排除前两年发生T2DM的参与者后，结果仍然一致。

结论: CCR/WC与中国成年人T2DM的风险呈负相关。对于具有低CCR/WC水平的中国人，早期检测对于控制T2DM的发展是必要的。

## 1 INTRODUCTION

Diabetes mellitus (DM) is a common public health problem with severe socioeconomic pressure. The International Diabetes Federation estimated the number of people with diabetes rising from 536.6 million (10.5%) aged 20–79 years in 2021 to 783.2 million (12.2%) in 2045.^1^ More than 90% of diabetes is type 2 diabetes^2^ (T2DM), leading to complications such as cardiovascular disease, stroke, renal failure, blindness, and disability.^3^, ^4^, ^5^ The diabetes death rate has also risen sharply, especially among older people and in less affluent areas.^6^ Therefore, early identification and timely intervention of people with diabetes are crucial.

Obesity is a vital risk factor for T2DM.^7^, ^8^ However, different from Western populations, people in Asia are more likely to develop T2DM with less weight gain and obesity.^9^ This difference is partially attributable to the loss of skeletal muscle mass, called sarcopenia.^10^, ^11^ What is worse, there may be a vicious cycle between sarcopenia and obesity; that is, sarcopenia reduces physical activity, increasing the risk of obesity, and obesity induces inflammation, leading to the development of sarcopenia.^12^, ^13^ Therefore, sarcopenia often coexists with obesity, called sarcopenic obesity^14^ (SO). SO is more likely to cause T2DM and metabolic syndrome.^15^, ^16^, ^17^ Consequently, to evaluate the T2DM risk, merely estimating obesity is insufficient and sarcopenia should be considered.

In terms of assessment of sarcopenia and obesity, magnetic resonance imaging, computed tomography, dual‐energy X‐ray absorptiometry, or bioelectrical impedance analysis are used in clinical practice. However, these methods are time consuming, inconvenient, and even cause radiation exposure. Thus, alternative, simple, and economical biomarkers are urgently needed for assessing sarcopenia and obesity. On the one hand, creatinine is the final product of the metabolism of creatine and phosphocreatine. At a steady state, the primary source of blood creatinine is skeletal muscle^18^ but its level varies with renal function. Cystatin C is produced by all nucleated cells and reflects glomerular filtration rate (GFR). Therefore, the creatinine to cystatin C ratio (CCR), known as a marker of muscle mass, was surrogated marker for sarcopenia.^19^, ^20^ On the other hand, the body mass index (BMI) is generally used to classify obesity.^21^ However, it cannot distinguish fat mass from lean muscle mass. Compared with BMI, waist circumference (WC) is a convenient measurement to assess body fat distribution and is more strongly associated with visceral adipose tissue.^22^, ^23^

Therefore, this study aimed to use the ratio CCR/WC as a surrogate for SO to investigate the association between SO and T2DM. Previous studies suggested that SO is strongly associated with a higher risk of T2DM.^15^, ^24^ Thus, we hypothesized that the ratio CCR/WC would strongly correlate with incident T2DM.

## 2 MATERIALS AND METHODS

### 2.1 Study population

This prospective cohort study was derived from the China Health and Retirement Longitudinal Study of 10 257 households and 17 708 middle‐aged and elderly participants from 150 counties or districts, 450 villages or urban communities in 28 provinces.^25^, ^26^ The respondents were followed up four times every 2 years through a face‐to‐face personal interview from June 2011 and March 2012 to July 2018 and March 2019. Moreover, the blood sample was collected in 2011 and 2015.

**FIGURE 1:**
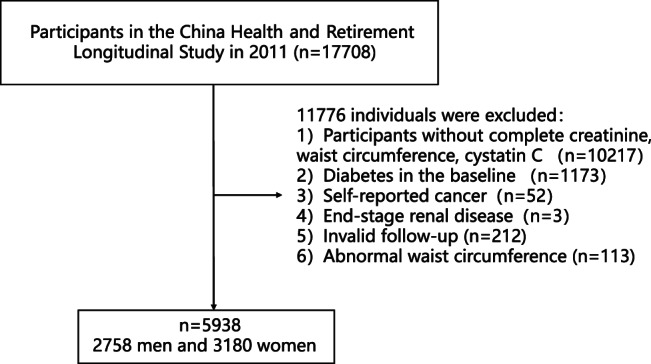
Flow diagram of the selection of participants.

Individuals with complete data on serum creatinine, WC, and cystatin C and who were not diabetic at baseline were included (*n* = 6318). We excluded those with self‐reported cancer (*n* = 52) and end‐stage renal disease^27^ (estimated GFR [eGFR] <15 mL/min/1.73m^2^) (*n* = 3). Participants who did not complete at least two visits were excluded (*n* = 212). We also excluded those with outliers of WC (<means minus 3 SD or > means plus 3 SD) (*n* = 113). Finally, a total of 5938 participants were included in the study (Figure 1).

The ethics review committee approved this study of Peking University. All participants signed written informed consent.

### 2.2 Measurement

At baseline, information on demographic characteristics, lifestyle, and medical history were collected by structured questionnaires. Smoking and drinking status were classified as never, former, and current. As for anthropometric measurement, body weight and height were measured to the nearest 0.1 kg and 0.1 cm, respectively, by light clothes and without shoes. BMI was calculated as weight (kg) divided by height (m) squared. WC was measured midway between the lower rib and the upper iliac crest to the nearest 0.1 cm. Systolic (SBP) and diastolic blood pressures were measured three times in the seated position after 10 min of rest by using a sphygmomanometer at intervals of 45 s, and the average was used for analysis. Professional nurses collected all venous blood samples after fasting for at least 12 h at night.^28^ Blood glucose, total cholesterol (TC), triglycerides (TG), low‐density lipoprotein (LDL‐C), and high‐density lipoprotein (HDL‐C) were measured by enzymatic colormetric test. The glycosylated hemoglobin (HbA1c) was measured by boronate affinity high‐performance liquid chromatography. eGFR was calculated from the creatinine level by using the Chronic Kidney Disease Epidemiology Collaboration (CKD‐EPI) equations.^29^ eGFR_CKD‐EPI_ was calculated as follows: for females with a plasma creatinine ≤0.7, (plasma creatinine/0.7)^−0.329^ × (0.993)^age^ (×144 if white or other); for females with a plasma creatinine >0.7, (plasma creatinine/0.7)^−1.209^ × (0.993)^age^ (×144 if white or other); for males with a plasma creatinine ≤0.9; (plasma creatinine/0.9)^−0.411^ × (0.993)^age^ (×141 if white or other); for males with a plasma creatinine >0.9, (plasma creatinine/0.9)^−1.209^ × (0.993)^age^ (×144 if white or other). The serum creatinine was tested by rate‐blanked and compensated Jaffe creatinine method. Cystatin C was measured by particle‐enhanced turbimetric assay. CCR was calculated as [creatinine (mg/dL)/cystatin C (mg/L)] × 100. CCR/WC ratios were calculated as creatinine to cystatin C to waist circumference.

### 2.3 Definition

Based on the American Diabetes Association criteria,^30^ T2DM was defined as fasting blood glucose ≥7.0 mmol/L, and/or random blood glucose ≥11.1 mmol/L, and/or HbA1c ≥6.5%, and/or self‐report diabetes, and/or taking antidiabetes treatment.

### 2.4 Statistical analysis

Baseline characteristics were described with median (interquartile range) for nonnormally distributed continuous variables and frequency (percentage) for categorical variables. The participants were classified by three CCR/WC tertiaries. Differences in baseline variables among the three categories were detected using analysis of variance for continuous variables and chi‐squared tests for categorical variables. Cox proportional hazards regression models were used to estimate hazard ratios (HRs) and 95% confidence intervals (CIs) for incident T2DM by tertiaries of CCR/WC, with the lowest tertiary as the reference. Model 1 was adjusted for age and sex. Model 2 was adjusted for model 1 plus marital status, educational level, smoking status, and drinking status. Model 3 was adjusted for model 2 plus BMI, SBP, TC, TG, LDL‐C, HDL‐C, and uric acid. *P* values for trends across CCR/WC tertiaries were calculated by a median value as a continuous variable. We used restricted cubic spline regression to explore possible linear or nonlinear correlation between incident T2DM and CCR/WC. Subgroup analyses were conducted by sex, age, and BMI. In the sensitivity analysis, first, we removed individuals who had T2DM within the first 2 years to control the potential contribution of reverse causation. Second, we used multiple imputations for missing data. Two‐sided *p* < .05 was considered as statistically significant. The statistical analyses were performed using SPSS software version 26 (SPSS Inc) and R statistical software version 3.6.1 (R Foundation).

## 3 RESULTS

### 3.1 Baseline characteristics of study participants

In total, 5938 participants (men = 2758, women = 3180) were involved in our analysis. The median age at baseline was 59.00 years and 53.6% were women. Table 1 shows the characteristics of the participants. According to CCR/WC tertiaries, participants were divided into three subgroups: tertiary 1 (Q1), CCR/WC ≤0.83; tertiary 2 (Q2), 0.84 ≤ CCR/WC ≤1.02; tertiary 3 (Q3), CCR/WC ≥1.03. Participants in higher tertiaries were more likely to be younger, male, married, high education level, alcohol drinkers, smokers, more likely to have lower hypertension, hyperlipidemia, and cardiovascular disease; were more likely to have higher levels of height, TC, HDL‐C, creatinine, uric acid; and were more likely to have lower weight, BMI, SBP, TG, LDL‐C, WC, and cystatin C. However, no significant difference in blood glucose, TG, and LDL‐C was found.

**TABLE 1:** Baseline characteristics of participants according to the tertiaries of CCR/WC ratios.

CCR/WC	Q1 (≤0.83)	Q2 (0.84–1.02)	Q3 (≥1.03)	*p* value^a^
Age (years)	62 (15)	59 (14)	56 (14)	<.001
Men	542 (27.40)	960 (48.50)	1256 (63.50)	<.001
Married	1601 (80.90)	1742 (88.00)	1780 (89.90)	<.001
Educational level^b^				
No formal education	1156 (58.4)	972 (49.10)	790 (39.90)	<.001
Primary school	785 (39.6)	943 (47.70)	1111 (56.10)	
Middle or high school	23 (1.2)	36 (1.80)	39 (2.00)	
College or above	14 (0.7)	28 (1.40)	39 (2.00)	
Smoking status^b^				
Never	1448 (73.10)	1170 (59.10)	987 (49.90)	<.001
Former	125 (6.30)	200 (10.10)	202 (10.20)	
Current	406 (20.50)	608 (30.70)	788 (39.8)	
Drinking status^b^				
Never	1397 (70.60)	1149 (58.10)	994 (50.20)	<.001
Former	154 (7.80)	210 (10.60)	192 (9.70)	
Current	427 (21.60)	620 (31.30)	791 (40.00)	
Hypertension, %	563 (28.40)	437 (22.10)	326 (16.50)	<.001
Hyperlipidemia, %	174 (8.80)	125 (6.30)	117 (5.90)	<.001
Cardiovascular disease, %	283 (14.30)	217 (11.00)	169 (8.50)	<.001
Height^b^, cm	154.90 (11.00)	157.60 (12.40)	160.00 (11.50)	<.001
Weight^b^, kg	58.50 (16.10)	56.60 (14.20)	55.90 (12.73)	<.001
BMI, kg/m^2^	24.14 (5.39)	22.86 (4.44)	21.81 (4.04)	<.001
SBP^b^, mm Hg	131.33 (29.33)	126.67 (28.67)	123.67 (25.33)	<.001
DBP^b^, mm Hg	75.00 (16.33)	74.00 (16.33)	74.33 (15.33)	<.001
PG, mg/dL	100.26 (14.40)	100.44 (15.48)	100.80 (15.66)	.581
TC, mg/dL	188.08 (50.64)	187.89 (46.01)	192.14 (46.78)	<.001
TG, mg/dL	105.32 (67.26)	100.01 (69.92)	99.12 (84.08)	.050
HDL‐C^b^, mg/dL	49.48 (18.17)	50.26 (19.72)	51.42 (20.49)	.001
LDL‐C^b^, mg/dL	115.59 (46.78)	114.43 (42.14)	113.66 (41.37)	.254
Uric acid, mg/dL	4.09 (1.45)	4.25 (1.55)	4.51 (1.71)	<.001
Creatinine, mg/dL	0.67 (0.18)	0.76 (0.20)	0.85 (0.23)	<.001
Cystatin C, mg/L	1.07 (0.29)	0.99 (0.24)	0.89 (0.24)	<.001
Waist circumference, cm	89.20 (13.40)	83.40 (11.90)	79.80 (11.90)	<.001

*Note*: Data were presented as n (%) or median with interquartile range.

Abbreviations: BMI, body mass index; CCR/WC, creatinine‐to‐cystatin C to waist circumference ratio; Cr, serum creatinine; DBP, diastolic blood pressure; HDL‐C, high‐density lipid cholesterol; LDL‐C, low‐density lipid cholesterol; PG, plasma glucose; SBP, systolic blood pressure; TC, total cholesterol; TG, triglycerides.

^a^ Comparison between Q1‐Q3.

^b^ Data for some participants were missing.

### 3.2 Risk of incident T2DM by tertiaries of CCR/WC

After 37679.00 person‐years follow‐up (median 7.00 years), 766 participants developed T2DM. Thus, the T2DM incidence was 20.33 per 1000 person‐years. Table 2 indicates the relationship between tertiaries of CCR/WC and T2DM incidence. With the CCR/WC increased, the incidence of T2DM declined in the crude and adjusted model. After adjusting for potential confounders, including age, sex, marital status, educational level, smoking status, drinking status, BMI, SBP, TC, TG, LDL‐C, HDL‐C, and uric acid. HRs (95% CIs) for T2DM across tertiaries of CCR/WC were 1.000 (reference), 0.772(0.647, 0.921), and 0.724 (0.596, 0.880; *p* for trend = .001).

**TABLE 2:** Incidence of T2DM by tertiaries of CCR/WC.

CCR/WC	Q1	Q2	Q3	*p* for trend
No. of subjects	1980	1979	1979	
Incident T2DM	316	234	216	
Incident T2DM (per 1000 person‐years)	25.86	18.40	16.95	
Crude	Reference	0.713 (0.602–0.844)	0.657 (0.553–0.782)	<.001
Model 1	Reference	0.753 (0.633–0.896)	0.729 (0.604–0.879)	.001
Model 2	Reference	0.757 (0.636–0.902)	0.734 (0.608–0.886)	.001
Model 3	Reference	0.772 (0.647–0.921)	0.724 (0.596–0.880)	.001

*Note*: Data are hazard ratios (HRs) and 95% confidence intervals (95% CIs). Model 1 was adjusted for age and sex. Model 2 was adjusted for age, sex, marital status, educational level, smoking status, and drinking status. Model 3 was adjusted as model 2 plus BMI, SBP, TC, TG, HDL‐C, LDL‐C, and UA at baseline.

Abbreviations: BMI, body mass index; CCR/WC, creatinine‐to‐cystatin C to waist circumference ratio; Cr, serum creatinine; DBP, diastolic blood pressure; HDL‐C, high‐density lipid cholesterol; LDL‐C, low‐density lipid cholesterol; PG, plasma glucose; SBP, systolic blood pressure; T2Dm, type 2 diabetes mellitus; TC, total cholesterol; TG, triglycerides; UA, uric acid.

**FIGURE 2:**
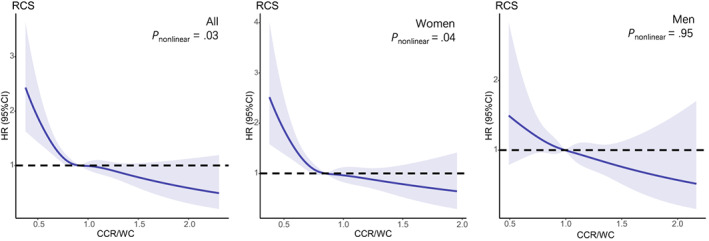
The nonlinear relationship between CCR/WC and incidence of T2DM. CCR/WC, creatinine‐to‐cystatin C to waist circumference ratio; HR, hazard ratio; RCS, restricted cubic spline; T2DM, type 2 diabetes mellitus.

**FIGURE 3:**
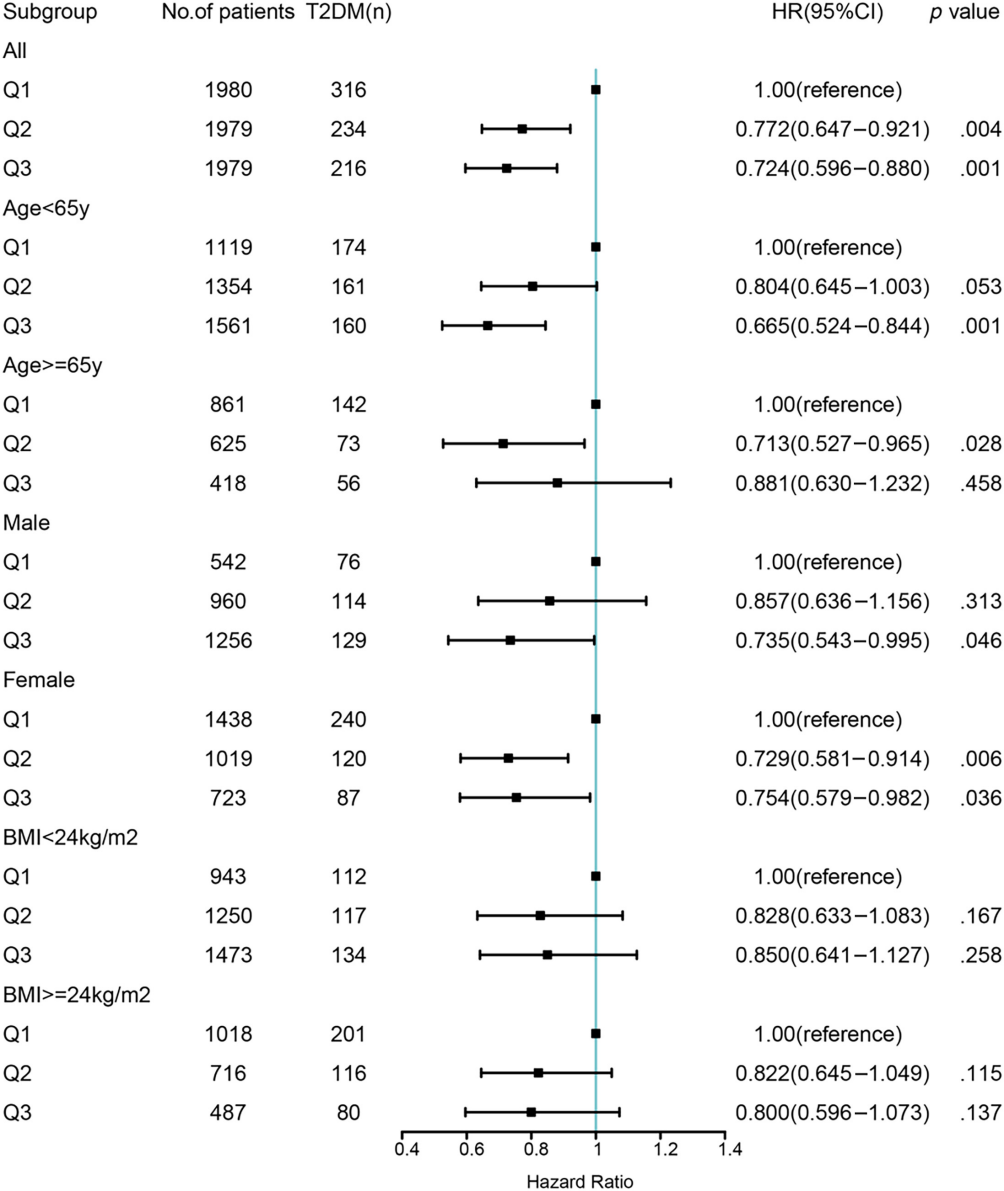
Association of T2DM and CCR/WC by age, sex, and BMI groups. Data are hazard ratios (HRs) and 95% confidence intervals (95% CI), adjusted for age, sex, marital status, educational level, smoking status, drinking status, BMI, SBP, TC, TG, HDL‐C, LDL‐C, and UA. BMI, body mass index; CCR/WC, creatinine‐to‐cystatin C to waist circumference ratio; HDL‐C, high‐density lipoprotein cholesterol; LDL‐C, low‐density lipoprotein cholesterol; SBP, systolic blood pressure; T2DM, type 2 diabetes mellitus; TC, total cholesterol; TG, triglycerides; UA, uric acid.

A nonlinear dose–response relationship was observed between CCR/WC and the risk of T2DM using restricted cubic spline regression (Figure 2). The HRs for the association between CCR/WC and incident T2DM decreased with increasing CCR/WC levels. The risk of T2DM decreased rapidly until around 0.91 of ratio and then started relatively flat afterwards (*p* for nonlinearity = .03). As CCR/WC elevated, the negative association between CCR/WC and the risk of T2DM became flatter when CCR/WC exceeded 0.85 for females. Further subgroup analyses of the ratio and incident T2DM were performed according to gender, age, and BMI (Figure 3). CCR/WC ratio was strongly associated with the incidence of T2DM in women but attenuated in men. We found at age < 65 years the HR (95% CI) for the association between CCR/WC ratio and T2DM incidence was 0.665 (0.524–0.844) and 0.804 (0.645–1.003) comparing Q3 and Q2 with Q1. In age ≥ 65 years, compared with Q1, a higher ratio was associated with reduced risk of T2DM in Q2 (HR, 0.713 [95% CI: 0.527–0.965]) and in Q3 (HR, 0.881 [95% CI: 0.630–1.232]). The relationship between the ratio and T2DM incidence is independent of BMI group.

**FIGURE 4:**
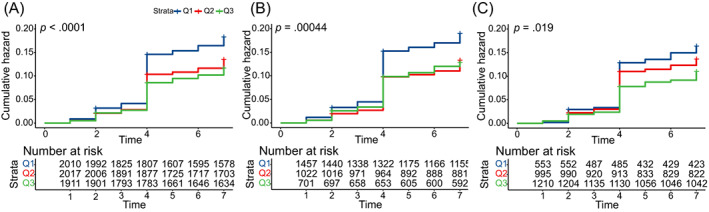
The cumulative risk of T2DM increased over time by this ratio (A). Moreover, it also found statistically significant differences in males and females respectively (B, C). In the female, the cumulative incidence of T2DM was significantly lower in subjects in higher tertiles of CCR/WC compared with those with the lowest tertile (*P* = 0.019 by log‐rank test).

The Kaplan–Meier curve indicated a significant difference in the incidence of T2DM among the CCR/WC tertiary (log‐rank test, *p* < .0001). The cumulative risk of T2DM increased over time by this ratio. Moreover, it also found statistically significant differences in males and females respectively (Figure 4).

### 3.3 Sensitivity analyses

We performed a sensitivity analysis to analyze potential confounders, shown in Tables S1 and S2. Excluding participants with impaired fasting glucose and T2DM in the first 2 years did not materially change the associations.

### 3.4 Multiple Imputation for Missing Data

The study contained 60 missing values of SBP, 30 of height, 29 of weight, 2 of education, 4 of smoking status, 4 of drinking status, 2 of HDL‐C, and 2 of LDL‐C. We observed the same consequence in tertiaries of CCR/WC and incident T2DM after multiple imputations for missing data, shown in Table S3. In model 3, HRs (95% CIs) for T2DM across tertiaries of CCR/WC were 1.000 (reference), 0.760 (0.638, 0.905), and 0.717 (0.591, 0.869; *p* for trend = .001).

## 4 DISCUSSION

This prospective cohort study demonstrated a negative correlation between the CCR/WC ratio and the incidence of T2DM in the Chinese population. The results were still consistent in adjusting for confounding factors and restricted cubic spline analysis showed a nonlinear dose–response relationship. Furthermore, CCR/WC ratio was associated with the incidence of T2DM in women but weakened in men through subgroup analysis. After multiple imputations for missing data or sensitivity analyses, the relationship between them did not change.

To the best of our knowledge, this is the first study to explore the association between the CCR/WC ratio and the risk of T2DM. CCR was a surrogate measure of muscle mass^19^, ^20^, ^31^ and was recognized as the sarcopenia index. The previous study showed that high normalized CCR is associated with reduced risk of T2DM, but did not consider the independent role of muscle mass and fat mass measures to assess T2DM risk.^32^ Consequently, the present study used CCR/WC to make a distinction between muscle mass, which may be protective against T2DM, and abdominal obesity, which confers increased risk for T2DM. In our series, the population with a high CCR/WC ratio had a lower risk of developing T2DM. The relationship is consistent with previous studies indicating that the balance of fat mass and muscle mass could be a considerable factor when assessing diabetes risk in the general population.^33^, ^34^ This seems to confirm that CCR/WC ratio is an excellent biomarker in our study population.

Our restricted cubic spline analysis also showed a reverse J‐shaped relationship between the CCR/WC ratio and the risk of T2DM. The improvements in CCR/WC ratio beyond 0.9 appear to yield less additional benefit in reducing the risk of T2DM, which is indicative of a threshold effect. However, our subgroup analysis indicated that the association between CCR/WC ratio and T2DM prevalence was statistically significant in women but attenuated in men. This difference may be explained by that men have more muscle mass than BMI‐ and age‐matched women.^35^ Alternatively, women have less muscle mass but more abdominal obesity than men. Therefore, the CCR/WC ratio of men is more easily affected by the threshold effect. However, further studies are needed to determine whether relationships between different body compositions and T2DM are influenced by sex. Interestingly, we found that different results for different age groups. Our results found that the median CCR/WC level in age < 65 years (0.85) was lower than that in age ≥ 65 years (0.95). We hypothesized that CCR/WC would reduce the prevalence of T2DM only if it reached a certain threshold. In addition, the number of participants diagnosed with T2DM in the age group older than 65 years is too few, which may be the reasons why the association weakened at age ≥ 65 years. Further research is needed on the distribution of sarcopenia in the elderly.

This relationship between the CCR/WC ratio and incident T2DM can tentatively be explained as follows. The skeletal muscle is the primary target organ of insulin‐mediated glucose disposal, accounting for 80% of glucose uptake.^36^, ^37^ Low muscle mass may decrease glucose utilization, causing hyperglycemia and increasing insulin resistance.^38^, ^39^ Therefore, people with sarcopenia, the age‐related decline in muscle mass, strength, and function, are susceptible to developing T2DM,^24^ nonalcoholic fatty liver disease,^40^ and metabolic syndrome.^41^ Conversely, reflected by a high WC, a high abdominal fat mass is the most important culprit responsible for insulin resistance.^42^ It has been shown that enlarged visceral fat cells secrete a number of inflammatory cytokines that lead to insulin resistance, such as interleukin‐6,^43^ and monocyte chemoattractant protein‐1.^44^ These factors worsen insulin resistance by triggering different key steps in the insulin‐signaling pathway.^45^ These cytokines can also stimulate the phosphorylation of serine residues in insulin receptor substrate‐1, thereby preventing the activation of insulin signaling and perpetuating insulin resistance.^46^

However, this study has several limitations. First, the initial design of the survey only had data from blood tests drawn in 2011 and 2015. No oral glucose tolerance tests were conducted in subjects, which may underestimate the risk of T2DM. Second, the survey did not address confounding factors such as diet and specific exercise levels. Third, we did not exclude patients taking drugs that affect renal function and muscle mass. Finally, the study was limited to the Chinese population and was not validated in other populations.

## 5 CONCLUSION

Our study demonstrated that CCR/WC negatively correlated with the risk of T2DM in Chinese adults. The lower the ratio, the greater the need for early screening for T2DM.

## AUTHOR CONTRIBUTIONS

Yinfei Chen and Zhiliang Mai contributed to the conception and design of the study, analysis and interpretation of the data, and drafted the manuscript. Weiheng Wen, Hong Chen, Jia Sun, and Ming Wang contributed to revising the manuscript and approved the final version. All authors read and approved the final manuscript.

## CONFLICT OF INTEREST STATEMENT

No potential conflict of interest relevant to this article was reported.

## Supporting information


**Table S1.** Excluding individuals who had diabetes in First 2 Years.
**Table S2.** Exclusion of participants with impaired fasting glucose (glucose between 6.1 and 7.0 mmol/L).
**Table S3.** Risk of T2DM by CCR/WC ratio based on multiply imputed data sets. CCR/WC, creatinine‐to‐cystatin C to waist circumference ratio; T2DM, type 2 diabetes mellitus.

## Data Availability

Data are from the China Health and Retirement Longitudinal Study (CHARLS) at http://charls.pku.edu.cn/ and can be accessed after application.
