# Supplementary material for: The serum creatinine to cystatin C to waist circumference ratios predicts risk for type 2 diabetes: A Chinese cohort study
Source: J Diabetes. 2023 Jul 5;15(10):808–16. doi: 10.1111/1753-0407.13435 (PMC10590677; doi:10.1111/1753-0407.13435)
Supplement: Supplementary file 1 — Table S1. Excluding individuals who had diabetes in First 2 Years. Table S2. Exclusion of participants with impaired fasting glucose (glucose between 6.1 and 7.0 mmol/L). Table S3. Risk of T2DM by CCR/WC ratio based on multiply imputed data sets. CCR/WC, creatinine‐to‐cystatin C to waist circumference ratio; T2DM, type 2 diabetes mellitus. [file JDB-15-808-s001.docx]

**Table S1** Excluding individuals who had diabetes in First 2 Years

| CCR/WC | Q1 | Q2 | Q3 | P for trend |
| --- | --- | --- | --- | --- |
| No. of subjects | 1917 | 1943 | 1932 |  |
| Incident T2DM | 253 | 198 | 169 |  |
| Model 1 | Reference | 0.788(0.651,0.954) | 0.704 (0.571,0.870) | =0.001 |
| Model 2 | Reference | 0.789(0.652,0.956) | 0.706(0.571,0.872) | =0.001 |
| Model 3 | Reference | 0.795(0.655,0.965) | 0.676(0.543,0.841) | ＜0.001 |

CCR, creatinine-to-cystatin C; WC, waist circumference;

Model 1 was adjusted for age and sex. Model 2 was adjusted for age, sex, marital status, educational level, smoking status, and drinking status. Model 3 was adjusted as model 2 plus BMI, SBP, TC, TG, HDL-C, LDL-C, and UA at baseline.

**Table S2** Exclusion of participants with impaired fasting glucose (glucose between 6.1 and 7.0 mmol/L)

| CCR/WC | Q1 | Q2 | Q3 | P for trend |
| --- | --- | --- | --- | --- |
| No. of subjects | 1640 | 1640 | 1621 |  |
| Incident T2DM | 215 | 162 | 151 |  |
| Model 1 | Reference | 0.780(0.633,0.962) | 0.777(0.620,0.974) | =0.027 |
| Model 2 | Reference | 0.785(0.636,0.969) | 0.790(0.630,0.990) | =0.039 |
| Model 3 | Reference | 0.800(0.646,0.990) | 0.773(0.612,0.977) | =0.029 |

CCR, creatinine-to-cystatin C; WC, waist circumference;

Model 1 was adjusted for age and sex. Model 2 was adjusted for age, sex, marital status, educational level, smoking status, and drinking status. Model 3 was adjusted as model 2 plus BMI, SBP, TC, TG, HDL-C, LDL-C, and UA at baseline.

**Table S3** Risk of T2DM by CCR/WC ratio based on multiply imputed datasets

| CCR/WC | Q1 | Q2 | Q3 | P for trend |
| --- | --- | --- | --- | --- |
| No. of subjects | 1980 | 1979 | 1979 |  |
| Incident T2DM | 316 | 234 | 216 |  |
| Model 3 | Reference | 0.760(0.638,0.905) | 0.717(0.591,0.869) | =0.001 |

Note: The models were adjusted for age, sex, marital status, educational level, smoking status, drinking status, BMI, SBP, TC, TG, HDL-C, LDL-C, and UA. Analyses were based on multiply imputed data sets including BMI, HDL-C, LDL-C, SBP, smoking status, drinking status, and education.
